# LuGSAM: a novel framework for integrating text prompts to Segment Anything Model (SAM) for segmentation tasks of ICU chest x-rays

**DOI:** 10.1007/s11042-025-21094-5

**Published:** 2025-09-17

**Authors:** Dhanush Babu Ramesh, Rishika Iytha Sridhar, Pulakesh Upadhyaya, Rishikesan Kamaleswaran

**Affiliations:** 1 Department of Electrical & Computer Engineering, Georgia Institute of Technology, Atlanta, Georgia USA; 2 Department of Biomedical Engineering, Georgia Institute of Technology, Atlanta, Georgia USA; 3 Department of Surgery, Duke University School of Medicine, Durham, North Carolina USA

**Keywords:** Chest X-rays, Grounding DINO, Object detection, Segment anything model, Lung segmentation

## Abstract

Segmenting lung regions in ICU Chest X-rays (CXR’s) is vital for diagnosing lung-related disorders, but existing methods require extensive annotations or training on large datasets. We present LuGSAM, a novel framework that integrates text prompts with the Segment Anything Model (SAM) for segmentation tasks, enhancing precision and adaptability in clinical settings. Our approach combines Grounding DINO, a zero-shot object detector using textual prompts (e.g., "right lobe"), and Meta AI’s SAM. Grounding DINO generates bounding boxes based on word-level prompts. These bounding boxes serve as an input to SAM, to generate precise segmentation masks. To further improve accuracy, we propose an iterative bounding box adjustment algorithm that refines object detections through multiple iterations. The Vision Transformer huge (Vit-h) variant of SAM achieved the highest overlap score (IoU = 0.95) for right lung segmentation. Grounding DINO demonstrated high detection accuracy for prompts like “right lung” with a confidence score of 0.58. The Binarized Predicted IoU (BPIoU) metric showed significant improvements in segmentation quality, making this framework a promising tool for clinical applications.

## Introduction

The development of foundational models has allowed for rapid advancements in medicine such as in medical imaging, drug discovery, and personalized medicine. While a number of Machine Learning (ML) and Deep Learning (DL) methods have been contributed for segmentation tasks, a general challenge is that such models are often trained for specific tasks. Training a DL model involves gathering a substantial amount of data and creating numerous ground-truth masks, leading to increase in number of annotation for the annotators [1]. One of the unmet needs in the field is the development of a versatile, all-in-one model that can perform segmentation across different tasks without requiring retraining or fine-tuning. Such models, often referred to as zero-shot learning models, hold the potential to revolutionize medical imaging by overcoming the limitations of task-specific models. In this direction, the SAM was introduced by Meta Research as a state-of-the art foundational model for segmentation tasks [2]. This benchmark model was trained on >1 billion masks and >1 million natural images and has broad applicability to various image segmentation tasks, including critical care settings like Intensive Care Units (ICUs) [3]. In an ICU setting, lung segmentation plays a crucial role in diagnosing and managing severe respiratory conditions such as pneumonia and Acute Respiratory Distress Syndrome (ARDS), which are common causes of respiratory failure and require precise imaging for effective treatment planning. Pneumonia, often complicated by ARDS, necessitates accurate lung imaging to assess disease progression and guide clinical interventions. The ability to segment lung images accurately can provide critical insights into the extent of lung involvement, aiding in the early detection of complications and improving patient outcomes. Hence in this study, we evaluate the performance of SAM using tailored anatomical text prompts on CXR images, primarily focusing on ICU CXR images which are crucial in emergency and critical care scenarios due to their complex nature. The motivation behind this work is to explore SAM’s ability to adapt to such challenging settings without requiring additional retraining or fine-tuning, making it a valuable tool for clinical application.

This research contributes to the broader field of Computer Aided Diagnosis (CAD), where significant advancements have been made in lung segmentation research. Segmenting infected nodules of the lung can provide comprehensive information about the type, location, and characteristic of the disease, which helps clinicians to better understand the progression of diseases, aiding in treatment planning [4]. This has led to the development of ML and DL based segmentation pipelines that focus on specific diseases with improved accuracy. However, as methodologies mature, these ML algorithms need to be rendered robust to support transition from bench to bedside [5]. In such cases, foundational models like SAM prove to be an all-in-one automated solution that can provide accurate segmentation masks with just a click on the Region of Interest (RoI) without having to train on domain specific data [6].

In our previous work, we used SAM to perform segmentation on CXR images, and found that SAM performed considerably well on segmentation tasks in terms of stability score, when prompted with points or bounding boxes. Stability score is a metric that calculates the differences in segmentation output of multiple perturbed images, given a single input image. The different types of segmentation like automatic, prompt-based, interface-based, and demographic based segmentation were explored and performed [7]. However, the text-prompt version of SAM has not been implemented yet and this work primarily focuses on providing customized anatomical text-prompts to SAM for segmentation of CXRs. While recent studies have examined SAM’s general performance in medical imaging, its potential in clinical scenarios such as lung segmentation remains underexplored [8]. Therefore, this study seeks to not only utilize SAM for interpreting CXR images but also to investigate the untapped potential of text-prompt-based segmentation. Furthermore, text prompts can guide AI models like SAM to focus on the desired regions of interest, which can result in efficient localization of objects. By using text-based cues, we aim to improve the model’s understanding of anatomical structures, thus reducing ambiguities in segmentation tasks by providing relevant clinical context.

In this work, text prompts were provided to the Grounding DINO model to identify the object in the image, and the detected bounding boxes were provided as prompts to SAM which produced the corresponding segmentation masks. The text prompts pertaining to the two lobes of the lungs were compared across the corresponding stability scores using histogram distribution plots. We found that word-level prompts resulted in more precise bounding boxes, thereby enhancing the overall segmentation accuracy. Additionally, it was observed that the most relevant prompts to the task yielded the best results in terms of bounding box precision. The contribution of this paper is summarized as follows:

- We introduce a novel framework that uses text prompts to guide bounding box detection in ICU CXR images.
- This framework is integrated into SAM to perform accurate lung segmentation, which can assist in disease detection and treatment planning.
- We enhance object detection using a binarization process on CXR images.
- An iterative bounding box adjustment algorithm is proposed to refine object detections.
- We compare the performance of SAM’s ViT variants using customized anatomical text prompts, demonstrating significant improvements in segmentation accuracy.

The rest of the paper is organized as follows. Section 2 reviews related work in the field of medical image segmentation. The proposed methodology, and architectural design is described in Section 3. Experimental setup including data used, evaluation metrics are presented in Section 4. The results are presented in Section 5. Finally, the discussion is presented in Section 6 followed by Conclusion and Future directions of the work.

## Related work

### SAM in medical imaging

SAM has shown great potential in the field of medical image segmentation. A study evaluated SAM’s ability to segment medical images across various datasets and modalities [9]. Trained on over 1 billion annotations, SAM’s performance was found to vary significantly based on the task and dataset. The results showed that SAM’s performance varied significantly depending on the task and dataset, with IoU scores ranging from 0.1135 for a spine Magnetic Resonance Imaging (MRI) dataset to 0.8650 for a hip X-ray dataset. The performance was higher for tasks involving well-defined objects with clear prompts and lower for more complex tasks like tumor segmentation. SAM’s performance improved slightly with multiple prompts, particularly for datasets where the object of interest was not contiguous. The study concluded that SAM has potential in medical imaging but requires further research for effective adaptation in this domain. Another similar study presented a tailored version of SAM for medical image segmentation, achieving competitive results in standard segmentation metrics. This SAMed applies a low-rank-based finetuning strategy to the SAM image encoder and is fine-tuned with the prompt encoder and mask decoder on labeled medical image segmentation datasets. The model achieves competitive results in both Dice Similarity Coefficient (DSC) and Hausdorff Distance (HD) metrics compared to existing methods in medical image segmentation, demonstrating its effectiveness in this specialized domain. These studies suggest SAM’s effectiveness in medical imaging [10].

### Deep learning-based segmentation

Deep learning techniques have made significant contributions to medical image segmentation. The paper "Deep learning ensemble 2D CNN approach towards the detection of lung cancer" utilized an ensemble of CNN models, showing a notable improvement in lung nodule detection from CT scans. The research uses the Lung Nodule Analysis (LUNA) 16 Grand challenge dataset, which consists of CT scans with annotations. The ensemble model combines different CNNs with various layers, kernels, and pooling techniques, resulting in a combined accuracy of 0.95, which is higher than the baseline method [11]. Similar to this, another study developed a model that detected lung cancer from chest radiographs. The model showed a sensitivity of 0.73 with a Mean False Positive Indication per Image (mFPI) of 0.13 in the test dataset. The study highlights the model’s ability to detect lung cancers with low mFPI, even in challenging cases where cancers overlapped with areas like the pulmonary apices, pulmonary hila, chest wall, heart, and sub-diaphragmatic space [12]. These studies underscore the efficacy of deep learning approaches in complex image segmentation tasks.

**Table 1:** Summary of Research Gaps in Related Work

Research Work	Method Used	Limitations	Research Gap	How LuGSAM Addresses the Gap
Mazurowski et al. (2023)	SAM on medical datasets with multiple prompts	Varies across tasks and datasets	Requires adaptation for complex tasks	Anatomical text prompts to guide SAM to focus on specific regions in ICU CXRs.
Zhang et al. (2023)	SAMed with low-rank fine-tuning	Requires fine-tuning	Need for an approach that leverages zero-shot learning	SAM’s zero-shot capabilities handle segmentation without fine-tuning.
Shah et al. (2023)	Ensemble CNN for lung nodule detection	Task-specific, CT-only	Lack of generalization to other conditions or modalities	Extends SAM to ICU CXRs without requiring condition-specific annotations.
Shimazaki et al. (2022)	Deep learning for lung cancer detection in radiographs	Limited accuracy, high false positives in complex cases	Need for more accurate segmentation	Text-prompt-based segmentation improves focus on relevant regions and accuracy.
Liu et al. (2022)	U-Net for automatic lung segmentation	Requires large annotated datasets	Dependence on large annotated datasets	Text prompts reduce the need for extensive annotation.
Portela et al. (2020)	DCNN with Dropout + L2 regularization	Task-specific, lacks generalization	Need for a generalized method for complex ICU settings	SAM provides generalization across ICU CXR segmentation tasks via zero-shot learning.

Note: SAM refers to the Segment Anything Model, and CXR refers to chest X-rays. This table summarizes key research gaps and the contributions of LuGSAM.

### Neural network-based segmentation

Neural networks, particularly in modified forms, have shown effectiveness in segmenting lung regions from CXR images. A study reported improvements in lung segmentation accuracy using a modified U-Net model. This paper discusses an improved version of the U-Net network for automatic segmentation of the lung region in CXR images. The modified U-Net uses the EfficientNet-b4 as the encoder and incorporates a Residual block and the LeakyReLU activation function in the decoder. The improved network is more efficient in extracting lung field features and avoids gradient instability during backpropagation. The modified U-Net model showed approximately 0.025 improvement in dice coefficient and 0.06 in Jaccard Index over the traditional U-Net model for benchmark lung segmentation datasets. The model demonstrated enhanced accuracy and robustness in lung segmentation from CXR images [4]. Another investigation into lung region segmentation with Deep Convolutional Neural Networks (DCNNs) explored three architectures combined with various regularization (Dropout, L2, and Dropout + L2) and optimization techniques (SGDM, RMSPROP, and ADAM). The most effective approach utilized Dropout + L2 regularization with the ADAM optimizer, achieving a high Jaccard Coefficient of 0.97967 ± 0.00232, surpassing state-of-the-art results. This method significantly reduced the time required for lung segmentation, making it an efficient tool for enhancing diagnostic processes in medical imaging [13].

The summary of the works conducted in this related field is presented in Table 1. The table addresses how LuGSAM attempts to bridge the gap in existing works.

## Methodology

### Proposed method

This section describes the proposed method for CXR segmentation using Grounding DINO and SAM. The proposed study was reviewed and approved by the Emory Institutional Review Board (IRB#STUDY00000302). The Algorithm 1 outlines the key steps involved in the process.

**Algorithm 1:**
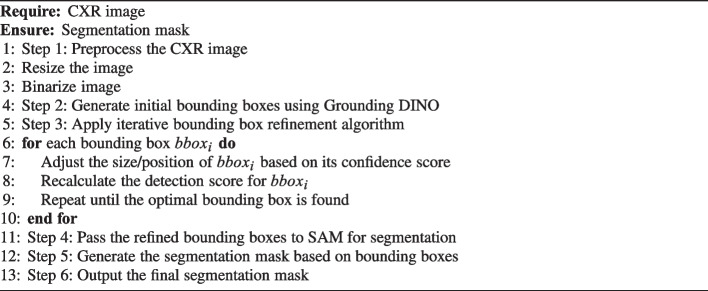
Proposed Method for CXR Segmentation.

### Architectural design

This section presents the architectural design of the two models utilized in our study: SAM and Grounding DINO. Both models, leveraging transformer-based architectures, complement each other for object detection and segmentation tasks.

#### SAM architecture

SAM consists of three main components: an image encoder, a prompt encoder, and a mask decoder. The image encoder is based on a Masked Auto Encoder (MAE) architecture and can process input images up to a resolution of 3x1024x1024. The backbone of SAM is the ViT, which comes in three variants—ViT-base (ViT-b), ViT-large (ViT-l), and ViT-huge (ViT-h) [3, 14]. This transformer backbone allows SAM to capture fine-grained details in images by splitting them into fixed-size patches that are linearly projected and assigned as tokens. Positional embedding provides location information for the patches, and the transformer mechanism enables SAM to capture long-range dependencies within the image [15].

The ViT architecture has been widely adopted in medical image segmentation tasks, such as those involving CT and MRI scans, where attention mechanisms play a crucial role in filtering irrelevant regions and highlighting salient features [16]. SAM’s attention mechanism, along with its zero-shot learning capability, allows it to generalize well to unseen images, making it suitable for real-time applications [17]. The model is optimized using focal loss and dice loss, and the AdamW optimizer is employed with $\beta _1$ = 0.9 and $\beta _2$ = 0.999. A linear learning rate warmup is applied for the first 250 iterations, followed by a step-wise learning rate decay schedule. Fine-tuning SAM on custom datasets or applying specific, relevant prompts can further enhance segmentation performance. The interplay between these optimization strategies and the multi-head attention mechanisms is critical to SAM’s robustness, hence we provide a mathematical formulation explaining how attention mechanism works in Appendix A.

SAM’s architecture further consists of encoder and decoder layers built upon the transformer backbone. The transformer architecture, particularly the ViT-huge, plays a crucial role in achieving effective segmentation without the need for fine-tuning and leverages the model’s zero-shot capabilities. The large layers are essential for capturing intricate patterns and long-range dependencies within high-resolution medical images, such as CXR’s. These images often contain subtle anatomical details that are critical for accurate segmentation. The depth of ViT-huge enables SAM to generalize across different datasets while maintaining high segmentation accuracy, particularly in critical medical scenarios where precision is vital.

#### Grounding DINO architecture

Grounding DINO is built upon the Detection Transformers (DETR) architecture, specifically the DINO model, and is capable of identifying and localizing objects in an image based on textual descriptions. It leverages a Swin transformer for visual feature extraction and BERT for extracting textual features. The model performs cross-modality fusion using a feature enhancer block with deformable self-attention to blend image and text features. Image-to-text and text-to-image cross-attention modules facilitate the fusion of visual and textual information, allowing the model to understand and align the image content with the text [18].

A language-guided query selection module helps select relevant features from the image based on the input text. The cross-modality decoder further refines these features using self-attention, cross-attention, and a feedforward network (FFN). Grounding DINO is trained with L1 loss and GIoU loss for bounding box regression, along with contrastive loss for aligning object and language tokens. This approach has enabled Grounding DINO to achieve an Average Precision of 63.0 on the COCO dataset.

Grounding DINO performs zero-shot detection, making it suitable for tasks where textual prompts are utilized to detect and segment objects. For instance, when given an image of a "scissor on a table," Grounding DINO can accurately identify and label both objects. The integration of Grounding DINO with SAM facilitates efficient cross-modality processing. Based on our understanding, the computational complexity can be approximated as $O(nm+nk)$, where *n* represents the number of pixels in the image, *m* denotes the text prompt length, and *k* is the number of detected objects. This approximation considers the cross-modality interaction in Grounding DINO and the mask generation process in SAM. While this provides a preliminary estimation, further analysis is required to validate the complexity.

**Fig. 1:**
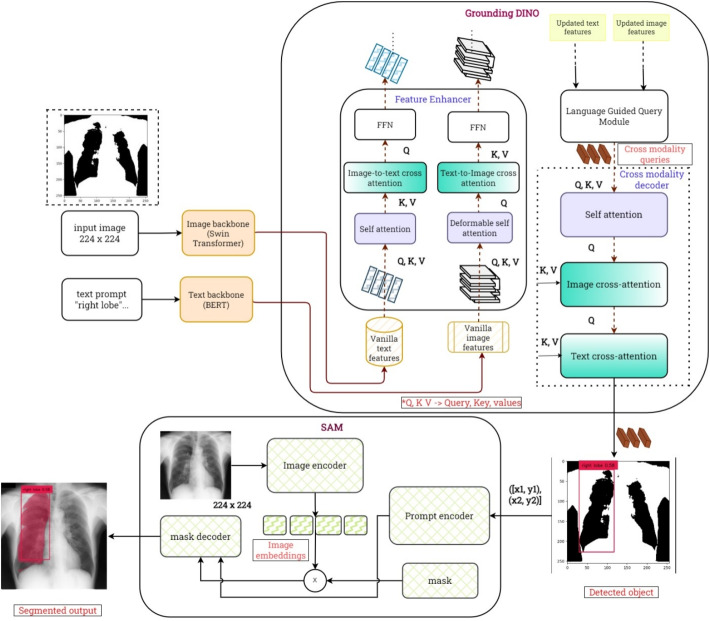
Complete pipeline of LuGSAM framework showing the integration of Grounding DINO and SAM architecture. The pipeline illustrates how image and text inputs are processed through respective backbones to generate bounding boxes, which then inform the segmentation process.

The overall architectural block diagram of the proposed work is shown in Fig. 1. This figure illustrates the architecture of Grounding DINO combined with SAM for lung segmentation. Grounding DINO uses a Swin Transformer to extract image features and BERT for textual prompts, which are passed through cross-attention and deformable attention layers to generate bounding boxes. These refined boxes are then processed by SAM, which further segments the lung region based on the text prompts.

**Table 2:** An analysis of the datasets and image sample used for the study indicating parameters like resolution, source of origin etc

Dataset	Source	Resolution	Images
Emory ICU	Emory University, High-resolution ICU images	3015 x 2505	216
Chest X-ray Masks	Montgomery & Shenzhen, TB and normal cases	4892 x 4020	215
NIH Chest X-ray	NIH Clinical Center, Diverse patient pool	1024 x 1024	200

## Experimental setup

### Data

**Fig. 2:**
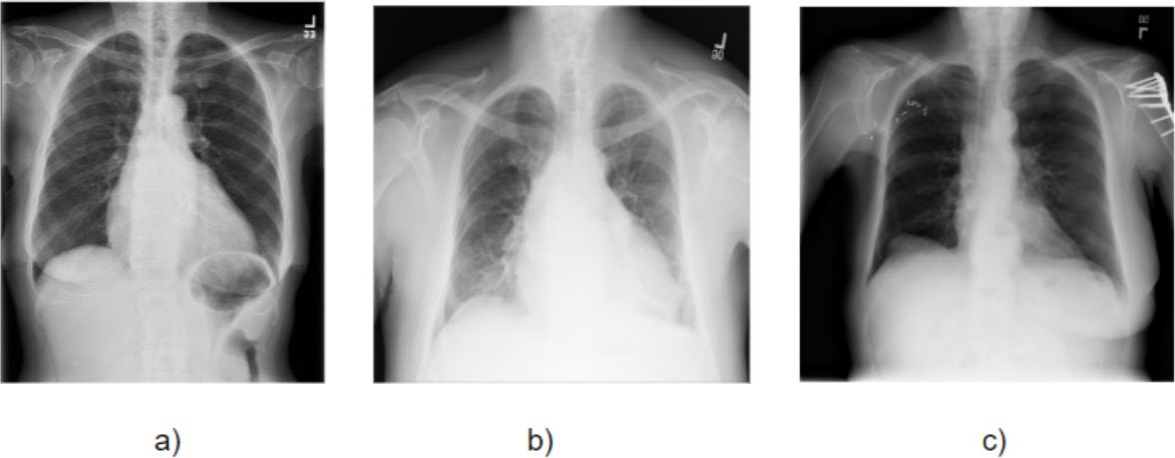
Sample images from the three datasets used in this study: (a) Emory dataset, (b) Chest X-ray Mask dataset, and (c) NIH Chest X-ray dataset. The images demonstrate variations in contrast, patient positioning, and anatomical coverage across datasets

- We obtained a total of 216 images from Emory for our study. The chest x-rays are predominantly single-view portable examinations. This type of imaging is often utilized in settings where patient mobility is limited, such as in ICUs or for bedridden patients. These images, were stored as Computed Radiography Image Storage with JPEG Lossless compression, and were captured with a pixel spacing of 0.10 mm, highlighting their high-resolution nature. The images dimensions were around 3015 x 2505 pixels, with a 12-bit storage depth, ensuring detailed visualization. The dataset originates from Emory University Midtown, utilizing the Fujifilm Corporation’s stationary X-ray machine. This dataset encompassed anonymized patient information, including demographic data and radiographic specifics (eg. View Position, Laterality etc.). All personal identifiers were removed to ensure patient privacy. Our analysis focused exclusively on the pixel arrays extracted from the chest X-rays. The associated metadata was not included in our analysis, and all data used was anonymized in accordance with IRB guidelines. The dataset is not publicly available and was accessed through Emory University’s internal research cluster.
- The "Chest Xray Masks and Labels" dataset, derived from the Montgomery County and Shenzhen chest X-ray datasets, includes X-rays and corresponding masks. The Montgomery County set, collected in Maryland, USA, comprises 138 X-rays with image dimensions of either 4020 × 4892 or 4892 × 4020 pixels, provided in 12-bit gray level PNG format, with optional DICOM format [19, 20]. It includes both normal cases and cases with TB manifestations. The Shenzhen set, gathered in China, consists of 662 X-rays of about 3000 × 3000 pixels, also encompassing normal and Tuberculosis (TB) cases. The dataset, useful for instance segmentation, semantic segmentation, and object detection tasks in the medical field, contains 896 images with 1627 labeled objects. These images have pixel-level instance segmentation annotations and are split into training and test sets. In our study we selected 215 single-view chest x-ray images from this dataset for dice loss analysis.
- To test the generalizability of our framework, we utilized diverse datasets including the NIH CXR dataset (100,000 de-identified chest X-rays, 1024×1024 pixels) and selected 200 single-view images from it. These images are accessible through the NIH download site [21]. Our study incorporates demographic diversity through the Emory dataset (urban academic medical center population, ages 18-89, balanced gender distribution), Montgomery County dataset (U.S. mid-Atlantic region), and Shenzhen dataset (southern China), encompassing various pathological conditions including critical illnesses like pneumonia, ARDS, and pulmonary edema from the Emory ICU dataset. Table 2 summarizes the datasets’ key characteristics while Fig. 2 shows sample images from each source, highlighting variations in image quality, contrast, brightness, and resolution—all factors significantly influencing segmentation accuracy.

### Model configuration

Both Grounding DINO and SAM were used as pre-trained models without any fine-tuning for this application. Since our approach leverages the zero-shot capabilities of these models, no training or data augmentation was performed. For Grounding DINO, we set the box threshold to 0.25 and text threshold to 0.25 based on preliminary experiments with a subset of our data. These threshold values provided optimal balance between detection sensitivity and precision. For SAM, we utilized the pre-trained weights of three model variants (ViT-b, ViT-l, ViT-h) as provided by Meta AI, maintaining their original parameter configurations. All inference was conducted using a single NVIDIA A100 GPU with batch size of 1 to ensure consistent processing across all images.

### Evaluation approach

Unlike traditional machine learning approaches that require dataset splitting into training and testing sets, our study utilized pre-trained models (Grounding DINO and SAM) in a zero-shot setting without any fine-tuning. This approach aligns with our research objective: to evaluate how foundational models can perform lung segmentation tasks without task-specific training. We evaluated the models’ performance directly on the complete datasets from three sources: Emory ICU (216 images), Chest X-ray Masks (215 images), and NIH Chest X-ray (200 images). Since no training or model optimization was performed, traditional train/test splits were not applicable. Instead, we assessed performance by comparing segmentation results against ground truth masks using established metrics across the entire dataset.

### Preprocessing

The following was the pre-processing method applied on the input CXR. The images obtained were resized to 224x224 for reducing computational complexity. The images were then converted to grayscale. Then, the images were fed to the Grounding DINO model for lung field detection. The box and text threshold were set to 0.25 and 0.25 respectively. To support multiple detections, irrelevant information from the images were suppressed by image binarization. This also helps in delineating the lung lobes from the background noises. Image binarization was done using OTSU thresholding with a threshold value set to 127 and the maximum value to 196. The binarization *B* for a threshold *T* with respect to an image *I* is expressed as in (1) where **B** is the binary image.

1 $$\begin{aligned} \text {B}(x_i, y_i) = {\left\{ \begin{array}{ll} 1, & \text {if } \text {I}(x_i, y_i) \ge \text {T} \\ 0, & \text {otherwise} \end{array}\right. } \end{aligned}$$

### Prompt framing

In this study, prompts for segmentation were framed in different ways, including individual words and complete sentences. Word-level prompts (e.g., "right lung", "left lobe") were found to consistently perform better in terms of segmentation accuracy. This superior performance can be attributed to several factors rooted in the architecture of transformer-based models. The attention mechanism in Grounding DINO allocates finite attention resources across all tokens in a prompt. Our analysis indicates that word-level prompts allow the model to concentrate its attention resources specifically on anatomical terms. In contrast, sentence-level prompts distribute attention across additional tokens (e.g., "segment," "identify") that do not directly contribute to anatomical localization. This attention dilution effect was consistently observed across our experiments. Furthermore, sentence-level prompts introduce potential semantic ambiguity that the model must resolve. For instance, in prompts like "segment the right lobe," the model must distinguish between instruction verbs and anatomical identifiers. This parsing complexity is entirely avoided with direct word-level prompts, allowing the model to focus on anatomical localization.

The following are examples of the prompt types that were tested:

- Word-level: "right lung", "left lobe"
- Sentence-level: "segment the right lobe", "identify both the lungs"

A detailed mathematical analysis of token-wise attention distribution and its effect on model performance is presented in Appendix B. This analysis provides a theoretical foundation for our empirical findings, demonstrating how attention weights become diluted when prompts contain irrelevant tokens. Based on these experimental results and theoretical analysis, word-level prompts were selected for the final model configuration.

### Evaluation metrics

To evaluate the performance of the segmentation models, we utilized several metrics that assess the overlap and accuracy of the predicted segmentation masks compared to the ground truth masks. The primary metrics used in this study were Intersection over Union (IoU), BPIoU, and Dice Loss. Each of these metrics provides a different perspective on the quality of the segmentation results.

#### Intersection over union (IoU)

IoU is a widely used metric to assess the quality of segmentation by measuring the overlap between the predicted and ground truth masks. It is defined as the ratio of the intersection of the predicted and ground truth masks to their union. IoU is particularly useful for evaluating how well the predicted segmentation aligns with the ground truth. The higher the IoU score, the better the segmentation performance [22].

#### Binarized predicted intersection over union

In addition to standard IoU, we introduced a custom metric called BPIoU. This metric is designed to assess the stability and accuracy of segmentation when the CXR images are binarized. The hypothesis is that higher IoU scores obtained after binarization indicate more accurate and stable segmentation masks. Conversely, lower BPIoU scores suggest poorer segmentation performance, indicating that the model struggled to produce precise masks.

BPIoU is calculated similarly to IoU but focuses on the binarized predicted mask $B(x_i, y_i)$ and the ground truth mask $G(x_i, y_i)$. The BPIoU metric is mathematically defined as:

2 $$\begin{aligned} B_{\text {PIoU}} = \frac{|B(x_i, y_i) \cap G(x_i, y_i)|}{|B(x_i, y_i) \cup G(x_i, y_i)|} \end{aligned}$$

#### Dice loss

Dice Loss was also used to quantify the overlap between the ground truth lung masks and the generated segmentation masks. The Dice coefficient, from which the Dice loss is derived, measures how similar the predicted and ground truth masks are by comparing their overlap. A Dice coefficient value closer to 1 indicates a better overlap, while a Dice loss value closer to 0 indicates minimal loss, i.e., better segmentation accuracy [23]. The Dice loss is computed as:

3 $$\begin{aligned} L_{\text {dice}} = 1 - \text {Dice Coefficient} \end{aligned}$$

The Dice coefficient is expressed as:

4 $$\begin{aligned} L_{\text {dice}} = 1 - \frac{2 \times \sum (M_{\text {ground}} \times M_{\text {generated}})}{\sum M_{\text {ground}}^2 + \sum M_{\text {generated}}^2} \end{aligned}$$

In the above equation, $M_{\text {ground}}$ represents the ground truth mask, and $M_{\text {generated}}$ represents the segmentation mask generated by the model.

**Table 3:** Table showing the object detection results for different prompts with the corresponding confidence scores

Prompt	Detected object	Comment
"right lung"		The prompt localized the right lung, achieving a confidence score of 0.26.
"right lobe"		The model detected both lobes of the lung with a confidence score of 0.29
"left lung"		The prompt identified both lobes, with a confidence score of 0.29
"left lobe"		The model exhibited difficulty in detecting the left lobe, with a confidence score below the threshold.

## Results

### Direct object recognition

Table 3 presents the performance of Grounding DINO when provided with various anatomical text prompts for lung segmentation. The model demonstrated variable detection capabilities across different prompts, with "right lobe" achieving the highest confidence score (0.29), while the "left lobe" prompt yielded significantly poorer detection results. When prompted with "right lung," the model detected not only the right lobe (confidence score: 0.26) but also recognized the left lobe (confidence score: 0.23). Similarly, both "right lobe" and "left lung" prompts successfully produced localized bounding boxes around their respective lung regions. However, the "left lobe" prompt resulted in inconsistent detections, indicating limitations in the model’s ability to interpret this specific anatomical reference. These findings establish a baseline understanding of Grounding DINO’s capability to recognize anatomical structures based solely on text prompts, without additional training on medical imaging data.

### Performance on binary images

The performance of Grounding DINO on binary images was evaluated. The results show the ability of DINO to concentrate only on the lobes of the lungs, which are the primary RoI, contributes to a large portion of its accuracy in binary image detections. DINO effectively isolated the lung structures from extraneous features by taking advantage of the binary representation, resulting in robust detections.

As observed in Table 4, prompts like "right lobe" accurately identified the RoI. The model was also provided with word-level prompts like "right . lung .", "left . lung .", resulting in accurate detections of RoI. However, the right lobe of the lung was identified with a confidence score of 0.41 when specified with the prompt "left . lung .".

**Table 4:** The detection performance of grounding DINO on binary images, detailing aspects about performance

Prompt	Detection	Comments
"right lung"		The prompt resulted in an unexpected identification of the left lobe, with a confidence score of 0.37.
"right lobe"		The model correctly identified the right lobe, with a confidence score of 0.45.
"left lung"		The prompt accurately detected the left lung with a confidence score of 0.37, showing reliable performance.
"left lobe"		The model incorrectly identified the right lobe with a confidence score of 0.42 when prompted for the left lobe.

### Grounding DINO + SAM

**Fig. 3:**
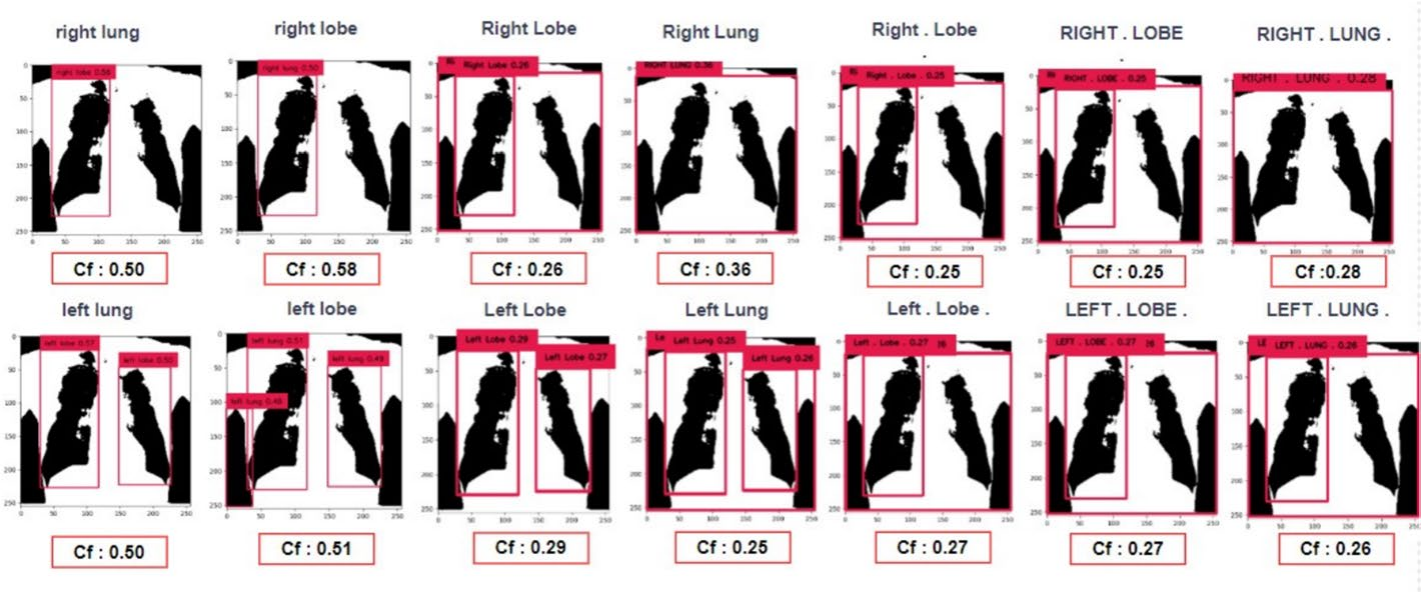
Grounding DINO results when prompted with the anatomical text prompts. Maximum confidence scores and accurate object detection are associated with the prompts "right lung", "right lobe", "left lung", and "left lobe"

**Fig. 4:**
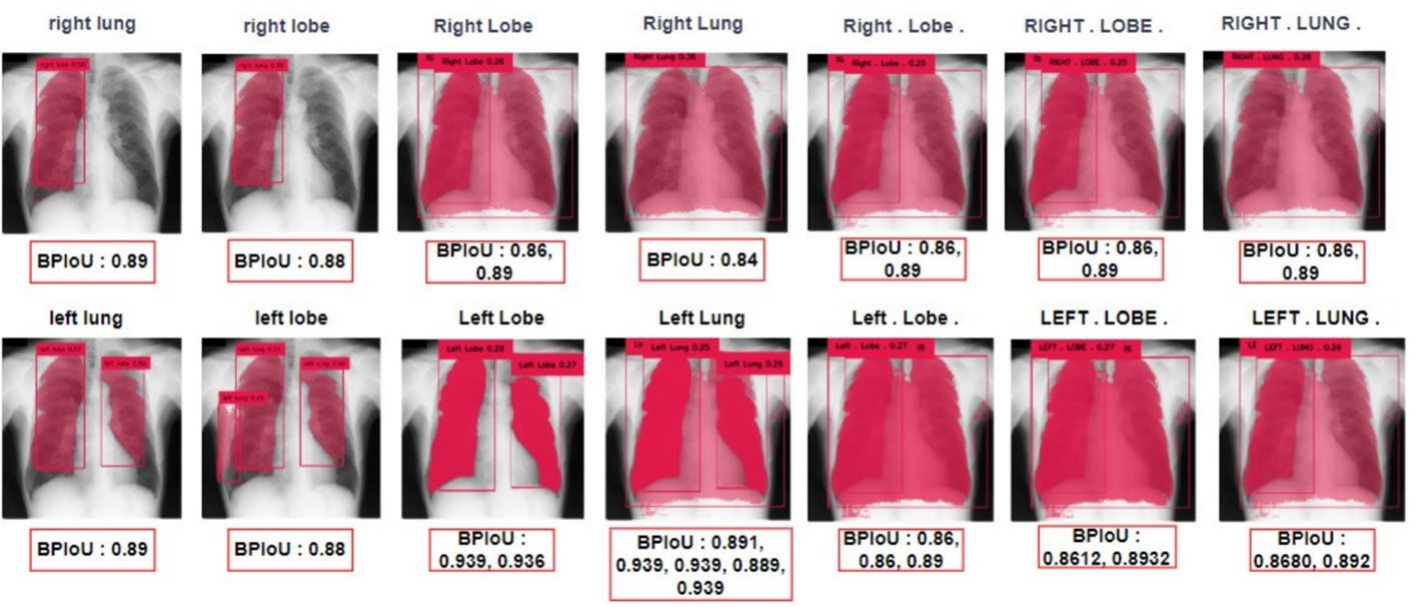
Results of SAM for the corresponding anatomical prompts. It can be clearly seen that the prompts "right lung", "right lobe", "left lung", and "left lobe" have achieved the best segmentation with a binary prediction over intersection score exceeding 0.89

The bounding box determined from the previous step was provided as input to SAM. The resulting segmentation masks from SAM were overlaid on the original image, forming Grounded SAM, which leverages the zero-shot learning capabilities of both Grounding DINO and SAM. Various prompts related to detecting the left and right lobes of the lungs were tested. The object detection and segmentation results for the right and left lungs are presented in Figs. 3 and 4, respectively. Figure 3 illustrates the confidence scores (Cf) for different text prompts, demonstrating better results for prompts like "right lung" with a Cf of 0.58. Figure 4 compares the BPIoU scores for segmentation results, with "right lung" achieving the highest BPIoU of 0.89, indicating accurate segmentation. Additional examples of challenging object detection scenarios are presented in Table 7 of Appendix C. These examples show how the system performs when dealing with poor image quality, cases involving implants, and other difficult detection conditions.

### Prompt ambiguity

**Fig. 5:**
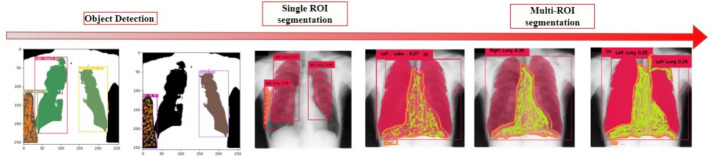
The figure shows ambiguous detections when prompted with lung anatomical prompts. The ambiguity begins in the DINO detection and propagates to successive stages. Similar issues are observed with single- and multi-ROI segmentations.

Figure 5 illustrates the challenge of prompt ambiguity in lung segmentation tasks, particularly when anatomical structures overlap or are nested within each other. Our analysis revealed that ambiguity initially occurs during the Grounding DINO detection phase and subsequently propagates through the entire segmentation pipeline, affecting both single and multi-ROI segmentations. When presented with ambiguous anatomical prompts, SAM generated multiple potential segmentation outputs, demonstrating its built-in capability to address uncertainty. This feature proves particularly valuable in clinical settings, as it allows healthcare professionals to select the most appropriate segmentation from several alternatives rather than forcing reliance on a single, potentially inaccurate result. The multiple-output approach effectively accommodates the inherent anatomical variation across patients while maintaining the interactive nature of the segmentation process.

**Fig. 6:**
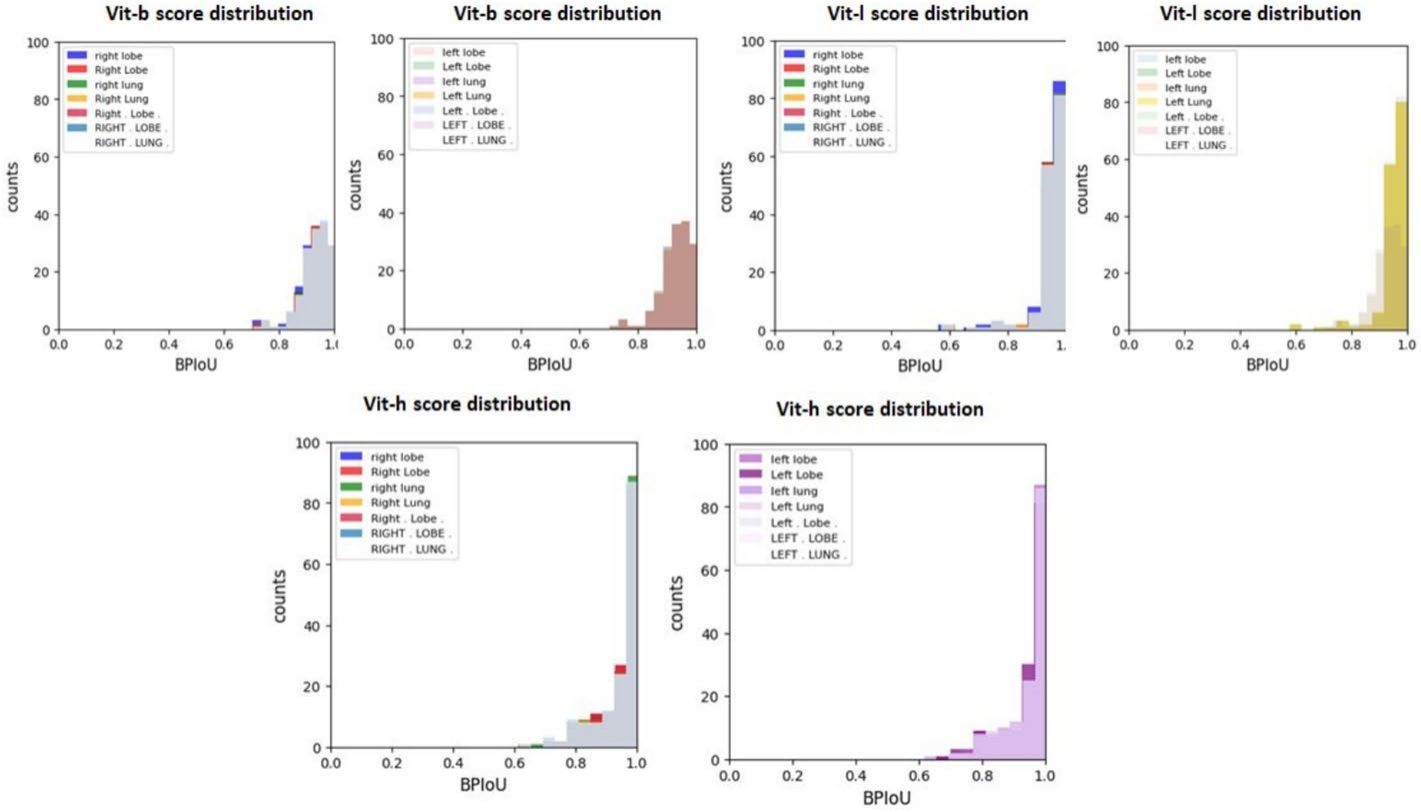
Histogram plots of scores obtained from various versions of SAM for the anatomical text prompts. Most of the results are clustered on the right side of the histogram. However, we can observe that Vit-h and Vit-l model has the best distribution. Similar results are seen for the prompts ("right lobe", "Right Lobe"), ("Right . Lobe .", "RIGHT . LOBE".) irrespective of letter capitalization. The same is observed for prompts pertaining to the left lung.

### Comparison of SAM versions

Figure 6 presents a comparative analysis of the three SAM variants (ViT-b, ViT-l, and ViT-h) through histogram distributions of BPIoU scores. The larger variants—ViT-h and ViT-l—demonstrated superior performance, with significantly more data points clustered in the 0.95-1.0 range compared to ViT-b. While ViT-b achieved respectable segmentation results (majority of scores within 0.8-1.0), it produced only approximately 40 high-performing segmentations, substantially fewer than its larger counterparts. Table 5 quantifies these performance differences through dice loss measurements, confirming the ViT-h model’s superior performance across all anatomical prompts. This variant proved particularly effective for left lung and left lobe segmentation, achieving dice loss values of 0.8024 and 0.8069, respectively. These findings indicate that the increased parameter count (636 million parameters in ViT-h versus 91 million in ViT-b) translates to measurable improvements in segmentation accuracy. As shown in Table 6, our LuGSAM method achieved a 95.0% IoU score for right lung segmentation, positioning it competitively among established lung segmentation approaches. This performance compares favorably with other state-of-the-art methods, including U-Net (95.1% on JSRT, 95.4% on MC) and various CNN-based architectures (Jaccard indices ranging from 88.0% to 98.5%), demonstrating the effectiveness of our approach for lung segmentation tasks.

**Table 5:** Dice loss of images with the corresponding ground truth masks. The Vit-h SAM variant achieves the maximum overlap with respect to all the prompts

	Vit-b	Vit-l	Vit-h
Left lung	0.7384	0.6991	0.8024
Left lobe	0.7486	0.7141	0.8069
Right lung	0.7351	0.6968	0.7990
Right lobe	0.7391	0.7105	0.7952

**Table 6:** Comparison of Jaccard Index (IoU) for different lung segmentation methods. It can be seen that LuGSAM achieves a jaccard index score of 95.0 %

Model/Method	Jaccard Index (%)	Dataset
CNN + Morphological Optimization [24]	98.5	JSRT
Atrous Convolutions [25]	96.1 (JSRT), 94.1 (MC)	JSRT, MC
Improved FCN [26]	95.8 (JSRT), 91.7 (MC)	JSRT, MC
U-Net [27]	95.1 (JSRT), 95.4 (MC)	JSRT, MC
AlexNet and ResNet [28]	88.0	MC
LuGSAM	**95.0 (Right Lung)**	Emory, MC, NIH

$^{1}$JSRT: Japanese Society of Radiological Technology dataset

$^{2}$MC: Montgomery County chest X-ray dataset

### Iterative Adjustment of bounding box prompts algorithm

**Fig. 7:**
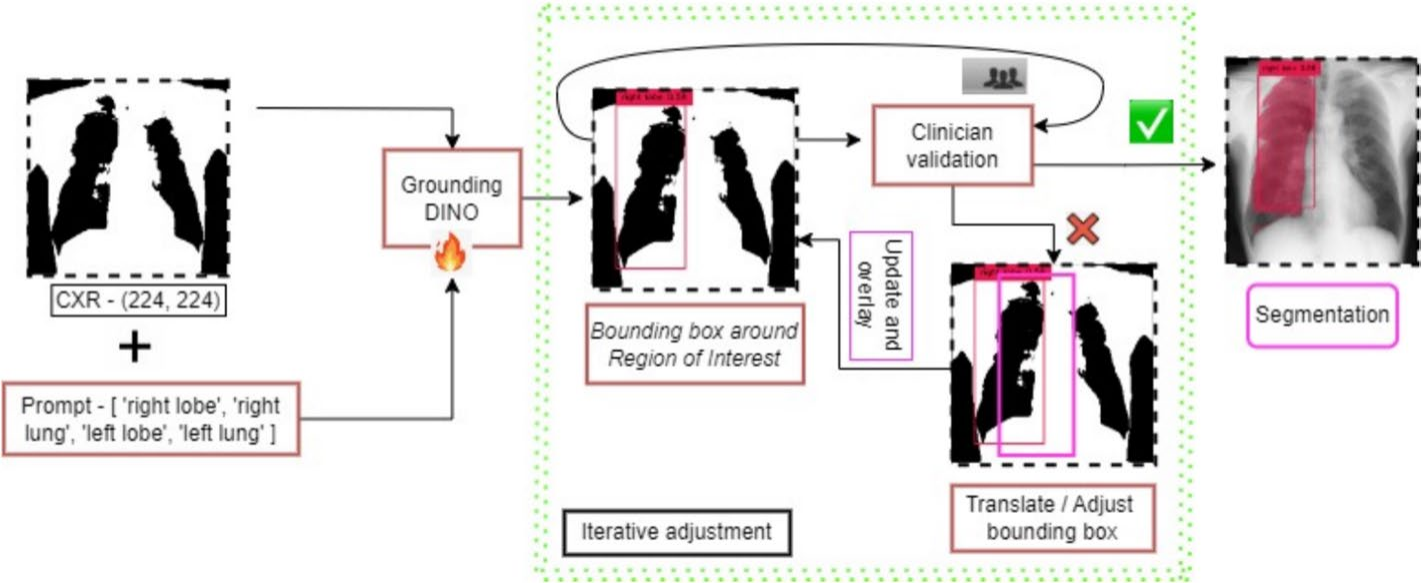
The iterative bounding box adjustment algorithm. The figure illustrates that given an input prompt and an image to the DINO model, a set of translations are applied such that the best segmentation is achieved

**Fig. 8:**
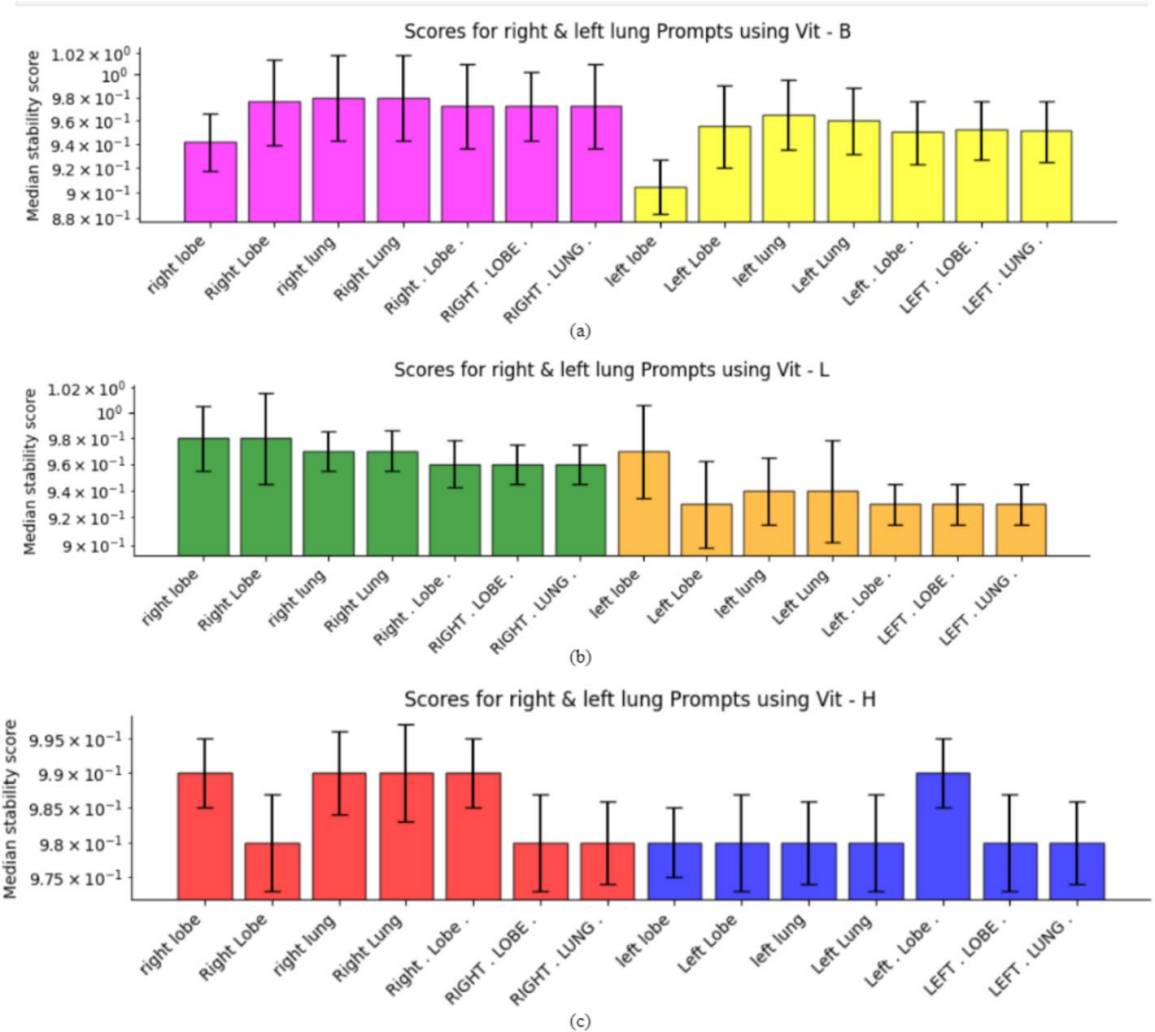
The figure panel represents three bar plots (a, b, c), each representing the performance of different Vision Transformer (ViT) models of SAM for lung region segmentation using various text prompts. The figure indicates consistent scores across all three variants of the SAM model specifically for prompts such as right lung, right lobe, left lung, and left lobe

To address the challenges observed with certain anatomical prompts—particularly those related to the left lobe—we developed an iterative bounding box adjustment algorithm, illustrated in Fig. 7. This algorithm refines the initial bounding boxes generated by Grounding DINO to achieve more accurate lung region segmentation. The implementation of this refinement process yielded significant improvements in segmentation accuracy, with confidence scores increasing by up to 0.21 in challenging cases such as "left lobe" detections. As shown in Fig. 8, the algorithm demonstrated effectiveness across all three SAM variants:

Figure 8(a) illustrates the performance of the ViT-b model, where median stability scores approaching 1.0 indicate successful refinement for both right and left lung prompts. The standard deviation bars reveal prompt-specific variability in performance. Figure 8(b) demonstrates that the ViT-l model achieved not only high median scores but also exhibited more consistent performance across different prompts, as evidenced by the shorter standard deviation bars compared to ViT-b. Figure 8(c) shows the ViT-h model’s performance, which maintained comparable consistency to ViT-l despite slightly lower median scores. These results confirm that our iterative refinement approach effectively addresses detection challenges in complex anatomical scenarios without requiring model retraining. The algorithm proved particularly valuable for challenging regions where initial detections were suboptimal, demonstrating a practical solution for enhancing detection reliability in medical imaging applications. The complete mathematical formulation of this algorithm is provided in Appendix C.

**Fig. 9:**
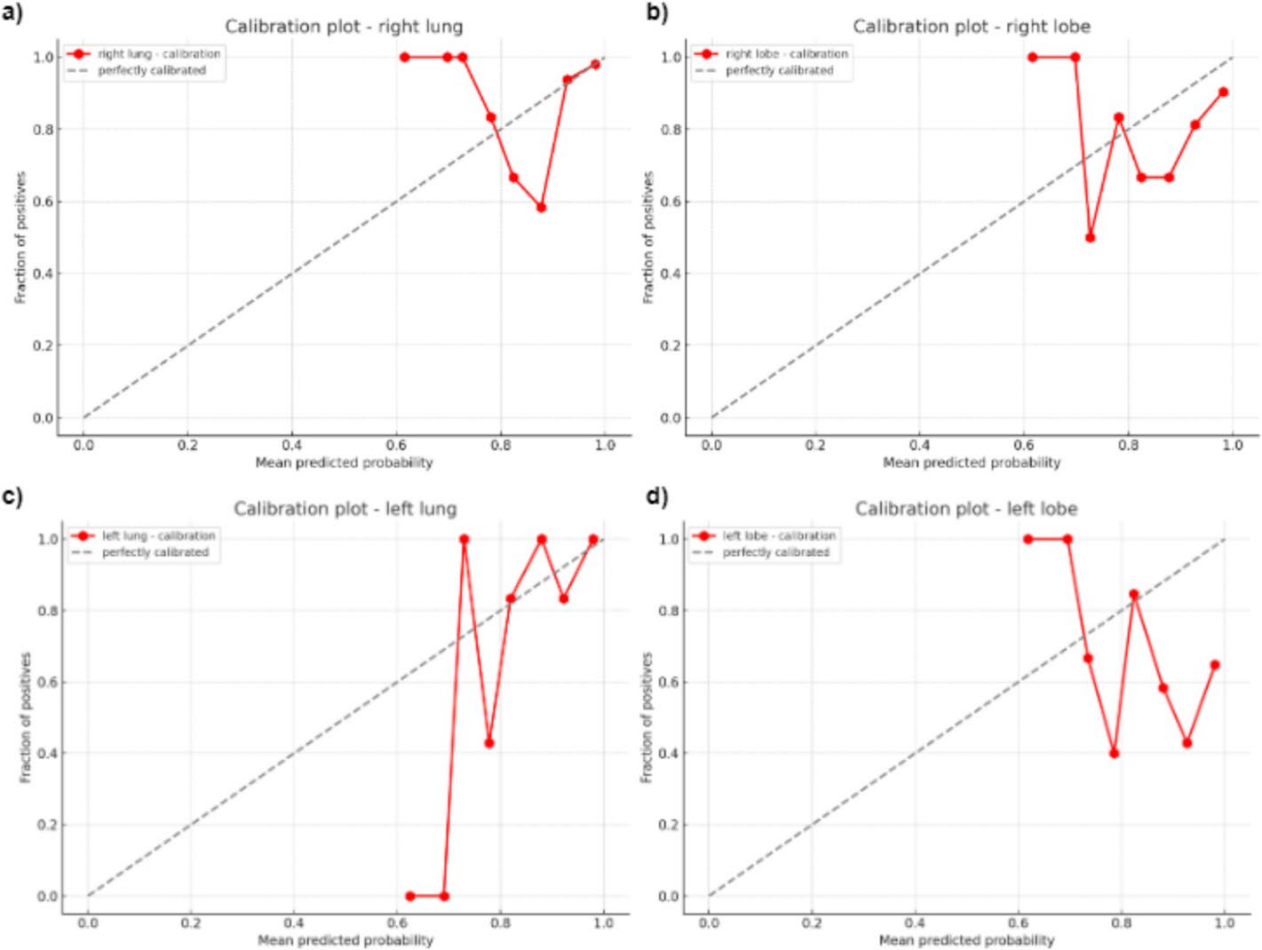
Prompt specific calibration plots for evaluating the model outcome

### Model evaluation

To further evaluate the performance of SAM, calibration plots were created. These plots were generated by converting the model’s IoU scores into a proxy for probabilities. This conversion is predicated on the assumption that there is a meaningful relationship between the stability scores and the probability of a good segmentation. Specifically, we interpret higher stability scores as indicative of a greater likelihood of a positive segmentation result, thereby allowing these scores to be scaled to a standard probability range (0 to 1). The assignment of ground truth values for evaluating SAM was binary. For each segmentation task, if the output of SAM closely matched the expected segmentation, indicating a high-quality segmentation, the ground truth was assigned a value of 1. Conversely, if the segmentation output significantly deviated from the expected result, demonstrating a poor segmentation quality, the ground truth was assigned a value of 0. This binary approach provided a clear and direct method to assess the accuracy of the segmentation tasks performed by the model. The calibration plots serve as a visual tool to assess how well these interpreted probabilities align with the actual outcomes. On the x-axis of each plot, we present the mean of these converted probabilities for grouped data points, delineated into bins. The y-axis represents the actual fraction of positive outcomes in each of these bins. The resulting calibration curve provides an intuitive visual representation of the SAM’s performance. In particular, the proximity of this curve to the diagonal line, denoting perfect calibration, is indicative of the model’s accuracy in predicting segmentation outcomes. For our study, calibration plots were generated for different prompts, such as "right lung," "right lobe," "left lung," and "left lobe" as shown in Fig. 9. For the "right lung" prompt (9(a)), the calibration curve closely approached the line of perfect calibration, with a majority of the data points clustering around the higher end of the probability scale. This indicates a high degree of alignment between the model’s output and the actual outcomes, underscoring the model’s effectiveness in accurately identifying the right lung region in CXR’s. Conversely, the plot for the "right lobe" prompt (9(b)), while still demonstrating a relatively good calibration, showed a slight deviation from the ideal line, particularly in the mid-range of the probability scale. This suggests a marginally lower but still substantial level of accuracy in segmentation for this specific prompt. The plots for the "left lung" and "left lobe" prompts further exemplified the model’s variable performance. The "left lung" plot (9(c)) depicted a calibration curve with moderate deviation from perfect calibration, particularly in the lower probability bins, indicating a good but slightly less consistent performance compared to the "right lung." In contrast, the "left lobe" plot (9(d)) exhibited more deviations across the probability spectrum, reflecting challenges the model faced in accurately segmenting this region, possibly due to anatomical and structural variabilities in the left lobe among different patients. These prompt-specific plots, therefore, not only highlight the overall effectiveness of SAM but also provide critical insights into its performance nuances, guiding future improvements and applications in medical image analysis.

**Fig. 10:**
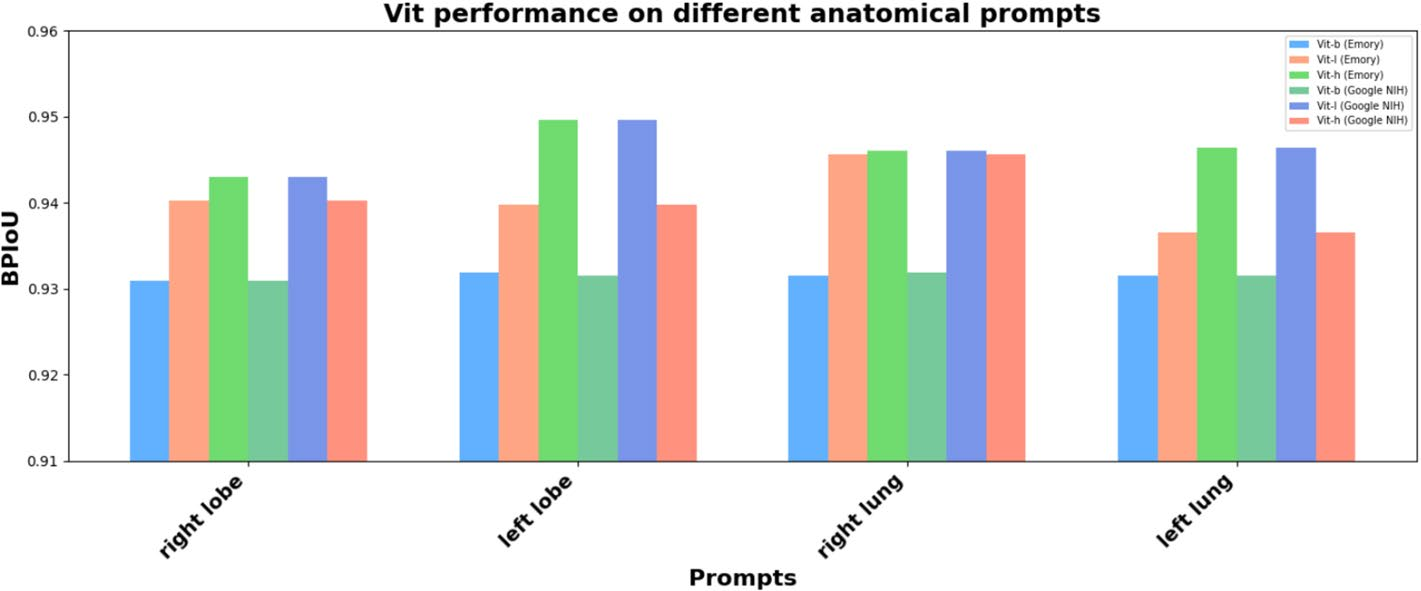
A comparison of the performance of three different ViT models for CXR image segmentation on data obtained from Emory and NIH clinical center. The first three bars in each set correspond to the EMORY dataset, while the following three bars represent data from the NIH clinical center. The metrics used was the BPIoU. Variations in BPIoU was seen when prompted with the four prompts indicated on the x-axis. It can be seen that Vit-l followed by Vit-h has achieved better results compared to the three other models. The mean BPIoU score for Vit-l for the prompts "right lobe," "right lung," "left lobe," and "left lung" on data obtained from Emory was 0.943044, 0.949624, 0.946081, and 0.946362 respectively. Similarly, the scores obtained for Vit-l on the NIH data was found to be 0.943044, 0.949624, 0.946081, 0.946362 respectively. The results show that the pipeline generalizes well and also suggest that Vit-l can be integrated with Grounding DINO to provide robust segmentation

## Discussion

The study demonstrates that the SAM model, combined with textual prompts, can effectively segment lung regions in chest X-rays. The ability to utilize text-based inputs offers a novel approach in clinical settings, providing a zero-shot method for segmentation without the need for extensive training or fine-tuning. Our findings show that simple word-level prompts like "right lung" or "left lobe" yield the most accurate segmentation results.

However, there are several factors that contribute to the variability in performance observed in this study. When CXR images were processed with Grounding DINO using the prompt "right lung," we obtained high-confidence detections. In contrast, prompts aimed at detecting the left lobe did not yield satisfactory results. This discrepancy could be attributed to variations in patient anatomy and the inherent complexity of chest X-rays, where tissue intensities often overlap due to the homogeneity of lung tissue.

Furthermore, we experimented with different types of prompts, including word-level and sentence-level variations. The results indicate that word-level prompts achieved higher accuracy, which we attribute to the reduced ambiguity compared to sentence-level prompts. Our analysis suggests that shorter prompts lead to more concentrated attention in the model, focusing on the critical components of the input text. A detailed elaboration of this can be found in Appendix B.

On the other hand, prompts such as "right lung" and "right lobe" achieved the highest BPIoU scores with confidence levels of 0.58 and 0.50, respectively. While sentence-level prompts such as "segment the right lung" did not perform well. So the variations in letter capitalization were tested, but they did not significantly affect performance. For example, prompts like "Right Lobe" and "RIGHT . LOBE ." achieved similar segmentation accuracy compared to the lowercase prompts. An interesting observation was that, the prompt "Left Lung" yielded multiple detections and identified the left lobe of the lung with an IoU of 0.93 indicating a very good segmentation. As a result, multiple bounding boxes were obtained and all the bounding box coordinates were given as prompts to SAM, which in turn performed multiple detections. IoU scores of 0.86, 0.89 were achieved for prompts "Right . Lobe .", and "RIGHT . LOBE .". Prompts with similar results were eliminated from further study and only select prompts were included in the performance analysis of text prompts on SAM, which is illustrated in Fig. 10.

The iterative bounding box refinement algorithm further improved segmentation results, particularly for difficult cases where initial detections were sub-optimal. This process involves dynamically adjusting bounding boxes based on confidence scores, enhancing detection accuracy across multiple iterations.

To ensure reproducibility of our experiments, we have provided a detailed description of the experimental setup in Section 4. Specifically, we used three datasets: Emory ICU, Montgomery & Shenzhen, and NIH Chest X-ray datasets, each with different imaging characteristics, such as resolution and pixel spacing. We applied preprocessing techniques including image resizing and OTSU binarization to standardize the inputs fed into Grounding DINO. The binarization method helped delineate lung structures, reducing the effect of noise from surrounding tissues.

We tuned several hyperparameters to optimize the performance of both Grounding DINO and SAM, including the box threshold (set to 0.25) and text threshold (also set to 0.25). These parameters were crucial in determining the quality of object detections and ensuring the best bounding box prompts were provided to SAM.

The iterative bounding box adjustment algorithm, described in detail in Appendix D, was crucial in refining detections, especially in cases where anatomical structures were not clearly distinguished in the initial detection.

Finally, the source code for this study, including the iterative algorithm and all related functions, is available on the Github repository.

## Strengths and limitations

The proposed method has several strengths. First, the use of text prompts, which can be quantitatively assessed, allows for a flexible and adaptive approach to object detection and segmentation without the need for extensive training on specific datasets. This zero-shot capability enables the model to generalize well to unseen images, making it highly versatile. Additionally, the iterative bounding box refinement algorithm strengthens the detection process by improving accuracy and precision without requiring fine-tuning, further enhancing segmentation performance in challenging cases and in low resource settings. However, one of the limitations of the study is that the effect of the proposed method on other imaging modalities, such as MRI or CT, has not been explored. This limits the generalizability of the method across different medical imaging types, which should be considered in future work.

## Conclusion and future works

This study delves into the segmentation of CXR images, widely used for their cost-effectiveness and capacity to diagnose lung disorders. We explored the potential of SAM to contribute to CXR image segmentation. SAM, with powerful object segmentation capabilities, was employed using text prompts through Grounding DINO—a zero-shot object detector. Grounding DINO was utilized to generate bounding boxes around objects, which in turn provided segmentation cues for SAM. Our research highlights the effective integration of Grounding DINO with SAM, particularly focusing on the ViT-h version, which demonstrated superior segmentation performance. This integration shows promise for applying SAM to medical image segmentation tasks, especially in critical settings like ICU CXR images. Although this study demonstrates SAM’s potential in medical image segmentation, further research is needed to address its limitations. With further development, LuGSAM could be adapted for real-time or automated diagnosis assistance, enhancing the efficiency and accuracy of clinical decision-making. In real-world clinical settings, such a system could aid radiologists by providing rapid, accurate segmentation of lung images, thereby facilitating quicker diagnosis and treatment planning. When addressing the potential of LuGSAM we also identified one direction for future work which is to optimize the prompt-engineering process to enhance segmentation accuracy in more complex clinical cases. Additionally, there is room to improve SAM’s efficiency when dealing with ambiguous or overlapping anatomical structures, a challenge often encountered in medical imaging. Another future focus could be evaluating SAM’s performance on a wider variety of medical imaging datasets, ensuring its robustness across diverse conditions. With these improvements and further research, SAM has the potential to make significant contributions to clinical diagnosis. The advancements in AI-driven medical imaging techniques, such as SAM, will continue to reshape the future of lung disease identification, ultimately improving patient care and healthcare outcomes.

## Figures and Tables

**Algorithm 2:**
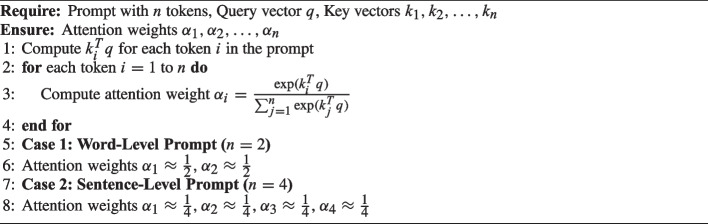
Attention Distribution Based on Prompt Length.

**Fig. 11:**
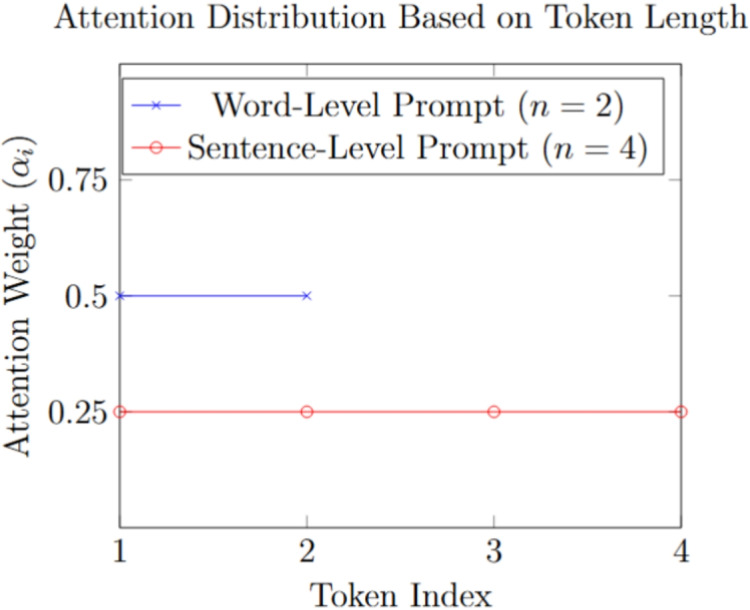
Effect of token length on attention distribution. Word-level prompts concentrate attention on fewer tokens, while sentence-level prompts distribute attention more evenly across anatomical and non-anatomical terms

**Algorithm 3:**
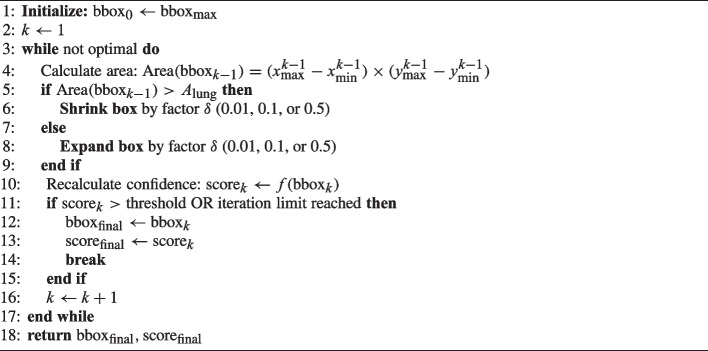
Iterative Bounding Box Refinement.

## Data Availability

The Emory ICU chest X-ray dataset used in this study is not publicly available and was accessed through Emory University’s internal research environment. All the data used in this study are available from the corresponding author upon request.

## Appendix A

### A.1 Attention mechanism

The attention mechanism in Transformer models is designed to capture dependencies in an input sequence by transforming token embeddings into query (Q), key (K), and value (V) vectors [29]. This process is enhanced with a masking procedure for applications such as language modeling, where future tokens should not influence the model’s predictions. The following steps outline this mechanism from an algorithmic standpoint which is crucial for understanding the Grounding DINO and SAM models since the core blocks of these models consist of attention.

#### A.1.1 Tokenization and embedding

When the model receives phrases like "right lung" and "left lung" it first breaks them down into individual tokens: ["right", "lung"] and ["left", "lung"]. Each token is then converted into a numerical vector (embedding) that represents its meaning. We denote these embeddings as $X = \{x_1, x_2,..., x_N\}$ where *N* is the number of tokens and each $x_i$ is an embedding vector in $\mathbb {R}^D$ with *D* being the embedding size.

#### A.1.2 Initialization of weight matrices

For each token embedding, the model computes:

- Query matrix $W^Q \in \mathbb {R}^{D}$ (Helps each token express what information it’s looking for),
- Key matrix $W^K \in \mathbb {R}^{D}$ (Helps each token express what information it contains),
- Value matrix $W^V \in \mathbb {R}^{D}$ (Represents the actual content each token contributes),

where *D* represents the size of the Q, K, and V vectors and *W* represents the weight matrix.

#### A.1.3 Computation of Q, K, and V vectors

For each token embedding $x_i$, in this case ["right"], ["lung"], we compute the *Q*, *K*, *V* matrices.

$$\begin{aligned} \text {Query: }&Q_i = x_i W^Q \text {(what this token is looking for), }\\ \text {Key: }&K_i = x_i W^K \text {(what information this token contains),} \\ \text {Value: }&V_i = x_i W^V \text {(Represents the actual content each token contributes)}. \end{aligned}$$

#### A.1.4 Mask initialization

To control which tokens can influence others, the model uses a mask matrix M. For language modeling, this mask ensures tokens can only attend to previous tokens (not future ones). This can be expressed as follows: Consider a mask matrix $M \in \mathbb {R}^{N \times N}$ which is defined such that $M_{ij} = 0$ if position *i* is allowed to attend to position *j*, and $M_{ij} = -\infty$ if not. For language modeling, *M* is a lower triangular matrix, ensuring that future tokens cannot influence current predictions.

#### A.1.5 Modified attention scores with masking

The attention scores are adjusted to account for masking as follows:

$$A'_{ij} = \frac{Q_i K_j^T}{\sqrt{D}} + M_{ij},$$

where $Q_i K_j^T$ measures the similarity between token $i$ and token $j$, $\sqrt{D}$ is a scaling factor to stabilize gradients, and $M_{ij}$ is the masking term to exclude irrelevant tokens.

The adjusted scores are normalized using the softmax function:

$$\alpha '_{ij} = \frac{\exp (A'_{ij})}{\sum _{k=1}^{N} \exp (A'_{ik})},$$

ensuring that the attention weights for each token sum to 1.

#### A.1.6 Output computation

The output for each token position is a weighted sum of all value vectors:

$$O_i = \sum _{j=1}^{N} \alpha '_{ij} V_j,$$

where $O_i$ represents the contextualized embedding for token $i$. This means each token’s final representation is influenced most by tokens with the highest attention weights.

## Appendix B

This appendix provides a theoretical analysis of why word-level prompts outperform sentence-level prompts in our segmentation tasks, focusing on attention distribution in transformer-based architectures like Grounding DINO.

### B.1 Mathematical foundation of attention distribution

In transformer architectures, the attention weight $\alpha _i$ for each token is determined by query-key similarity. Given a query vector $q \in \mathbb {R}^d$, value vectors $\{v_1, v_2, \dots , v_n\}$, and key vectors $\{k_1, k_2, \dots , k_n\}$ (where each vector $\in \mathbb {R}^d$), the attention weight for token *i* is computed as:

5 $$\begin{aligned} \alpha _i = \frac{\exp (k_i^T q)}{\sum _{j=1}^n \exp (k_j^T q)} \end{aligned}$$

This equation demonstrates that attention is a mechanism where tokens compete for finite attention resources, with the total attention summing to 1 across all tokens.

### B.2 Token length impact on attention

When using sentence-level prompts (e.g., "segment the right lung"), the number of tokens *n* increases significantly compared to word-level prompts (e.g., "right lung"). This increase directly impacts attention distribution:

In the idealized case, attention is evenly distributed, but real-world scenarios often show uneven distributions. With sentence-level prompts, non-anatomical tokens like "segment" may receive substantial attention, diverting focus from anatomical terms critical for segmentation.

### B.3 Visualization of attention distribution

Figure 11 illustrates this effect visually. With word-level prompts (n=2), attention is concentrated on the anatomical terms. In contrast, sentence-level prompts (n=4) distribute attention across all tokens, including non-anatomical ones, diluting the focus on key anatomical information.

### B.4 Implications for lung segmentation

This attention dilution directly correlates with our empirical findings in Section 4.5. When Grounding DINO processes sentence-level prompts, attention becomes distributed across more tokens, reducing the model’s focus on critical anatomical terms. Consequently, the model generates less precise bounding boxes for anatomical regions, which in turn affects SAM’s segmentation accuracy.

Our experimental results consistently showed that word-level prompts like "right lung" produced more accurate bounding box detections compared to equivalent sentence-level prompts like "segment the right lung." This theoretical analysis explains why simpler, more focused anatomical terms yield superior segmentation performance in our LuGSAM framework.

## Appendix C

Table C1 demonstrates the performance of the Grounding DINO model when prompted with "right lung" across different chest X-ray (CXR) images of varying quality. The table consists of five test cases showing both the original images (left column) and their corresponding detection results (middle column) with colored bounding boxes indicating the model’s predictions. The results reveal that image quality significantly impacts detection accuracy. Despite poor image contrast in some cases (rows 1-2), the model still identifies lung lobes with moderate confidence scores (0.57 and 0.53). In cases with poor alignment or multiple detections (rows 3-5), the model sometimes focuses on incorrect regions or produces multiple candidate boxes, though it correctly identifies the right lobe in most instances. This highlights that proper image alignment, contrast, and quality are critical factors for optimal performance of the Grounding DINO model in lung segmentation tasks. This analysis supports our approach of using anatomically-specific text prompts in the LuGSAM framework, while emphasizing the importance of image preprocessing for reliable detection results.

**Table 7:** The performance of the grounding DINO model on a few raw and binarized images for the prompt "right lung"

Image	Detection	Comments
		The prompt identified the left lobe with a confidence score of 0.57 since the image is very poor.
		The prompt identified the right lobe with a confidence score of 0.53 despite poor contrast in the original image.
		The prompt for this image resulted in multiple detections focusing on the left side of the CXR.
		The prompt has identified the right lobe the lung with a confidence score of 0.43 which highlights the importance that CXR should have proper alignment and contrast.
		Even though multiple detections are resulted from the prompt, the right lobe is correctly identified with a confidence score of 0.43.

It can be seen that the detections improve with the quality of CXRs

## Iterative bounding box algorithm

This appendix provides the mathematical formulation and detailed implementation of the iterative bounding box adjustment algorithm described in Section 5.6.

### Mathematical formulation

Let $D = \{d_1, d_2, \ldots , d_n\}$ be the set of all detections from Grounding DINO, where each detection $d_i$ consists of:

- A bounding box $\text {bbox}_i = (x_{\text {min}}^i, y_{\text {min}}^i, x_{\text {max}}^i, y_{\text {max}}^i)$, representing the coordinates of the top-left and bottom-right corners
- A confidence score $\text {score}_i$ representing the model’s certainty about the detection

We begin with the highest-confidence detection:

6 $$\begin{aligned} d_{\text {max}} = \underset{d_i \in D}{\arg \max }\ \, \text {score}_i \end{aligned}$$

This gives us the initial bounding box $\text {bbox}_{\text {max}}$ which serves as the starting point for refinement.

### The Refinement Algorithm

The iterative refinement process addresses limitations in initial detections, particularly for complex anatomical structures like the left lung lobe. The algorithm proceeds as follows:

In this algorithm, $A_{\text {lung}}$ represents the expected lung area (approximately 2000 pixels in our 224×224 resolution images). The algorithm uses three translation factors (0.01, 0.1, and 0.5) that are applied to the dimensions of the bounding box, allowing for both fine and coarse adjustments.

The box is expanded or contracted based on its current size compared to the expected lung area. For expansion, dimensions increase outward by the factor $\delta$ multiplied by the current width/height. For contraction, dimensions decrease inward by the same calculation.

After each adjustment, the confidence score is recalculated. The process terminates when either:

- The confidence score exceeds a threshold (0.25 in our implementation)
- A maximum of 10 iterations is reached
- The confidence improvement between iterations falls below 0.01

This refined bounding box is then passed to SAM for final segmentation, as described in Section 5.6 of the main text. This approach significantly improved detection accuracy, particularly for the left lobe, which initially showed lower confidence scores.

## Abbreviations

Abbreviation: Description
CXR: Chest X-ray
PII: Patient Identifiable Information
ICU: Intensive Care Unit
SAM: Segment Anything Model
GD: Grounding DINO
BERT: Bidirectional Encoder Representations from Transformers
IoU: Intersection over Union
BPIoU: Binarized Predicted Intersection over Union
ViT: Vision Transformer
MAE: Masked Autoencoder
CNN: Convolutional Neural Network
DSC: Dice Similarity Coefficient
OTSU: Otsu’s Binarization Method
LUNA: Lung Nodule Analysis
HD: Hausdroff Distance
MC: Montgomery County
MRI: Magnetic Resonance Imaging
CT: Computed Tomography
